# Hospital Economics

**Published:** 1900-12-15

**Authors:** 


### The Institutional Workshop.

### HOSPITAL ECONOMICS.

By a Correspondent.

"How to Lessen Coal Bills: by Adapting an Ordinary Wasteful Grate, at a Small Cost, to give more Heat with less Fuel: with some Suggestions on the Economy of Fuel for Domestic Purposes generally." By Francis T. Bond, M.D., B.A.Lond., F.R.S.Ed., Medical Officer of Health, Gloucestershire Combined District.

This is the title—a very attractive one at the present time —of a pamphlet, published by the Sanitary and Economic Association, Limited, Gloucester, which we have received. It was first published fifteen years ago, when coal was dear, and therefore it is not remarkable that some of the suggestions contained in it have been already adopted. But it is undeniable that we, as a nation, still waste much of the heat of the coals we burn, and think of economy only when a sudden rise in the price of coal upsets our household bills. Among those of Dr. Bond's suggestions which have been more or less accepted by the British public since first he wrote on the subject may be mentioned that of the slow-combustion grate. It is true, as he says, that a slow-combustion grate does not necessarily give greater heat than an ordinary one, but by the mere fact of slow combustion it does not send the heat up the chimney so fast. As we have no desire to heat the outer air, this is a merit; but it must be admitted that in some slow-combustion grates the heat thrown out is insufficient. Sometimes—as, for example, when a fire is first lighted—a greater draught is required than when the fire is well established. Therefore, he recommends the closing of the space under the fire by a close-fitting but movable front. This is found in most of the modern slow-combustion grates. So also is another of his suggestions, the reducing of the depth of the fire basket to not more than six inches, while keeping it as wide as ever in front, to give the greatest possible surface for radiation. For making the depth less he advises the use, not of a solid firebrick, but of a perforated one. The perforation should slope from behind upwards and forwards, so as to conduct air, heated as it passes through, into the body of the fire. The air will help to make the fire burn, without taking too much of its heat in return, as happens when we allow a draught of cold air to enter the fire from the front. As this draught also incommodes those who occupy the room it is doubly objectionable.

Dr. Bond is strongly in favour of the use of gas as fuel, but does not think highly of the way in which it is generally used. The gas and asbestos fire he regards as extravagant and noxious. Not only does the best part of the heat go up the chimney, but, from the way in which the gas is burned, a good deal of an especially noxious gaseous product (*acetylene*) is formed, which is very apt to escape into the room and to render its atmosphere offensive, depressing, and even dangerous. He therefore recommends the use of gas in conjunction with coke or anthracite. "There is thus not only no waste of the heating power of the gas, but a positive economy of that of the fuel, which is thus burned under the most favourable conditions." It is impossible to burn either coke or anthracite in an open firebasket without so great a draught as to be extravagant, but with gas to light it, it is easy and convenient. The present writer uses this combination of gas and anthracite or a patent smokeless fuel known as "paraffin coke" and finds the combination convenient, cleanly, and economical. The gas can be extinguished after the coke is thoroughly lit, and unless the fire is allowed to get very low need not be used again all through the day. If needed, however, it is always at hand, and is quick and sure in its action. Indeed Dr. Bond's recommendation to use gas instead of wood or fire-lighters to light even an ordinary coal-fire has much to recommend it.

It is, however, easier and cleaner to use gas alone, and Dr. Bond is of opinion that to this end no elaborate contrivance is needed. "Any ordinary burner," he says, "so protected that it cannot set fire to combustible material that may come into its neighbourhood, and placed as low as possible on the floor of the room, will give better results than many of the much more costly delusions which are so abundantly foisted on the public for the purpose." It must be admitted, however, that the average Briton does not take kindly to a gas-fire. It does not seem to him the genuine article. He likes to see a fire, and to be able to poke it, if his feelings require to be relieved in that way. It is doubtful if he would even take kindly to the American idea of burning anthracite in a closed stove with mica windows through which the glow of the fuel can be seen. And it is to be feared that he will never take to the closed stove for domestic heating, although its obvious economy should cause it to be more generally adopted in halls, offices, and other places where physical warmth is desired without those sentimental associations which surround the idea of the fireside. Even so, he may reduce the coal-bill in several ways. One is to combine the use of coke with coal in an ordinary fire. The fire cannot be first made with coke, but if mended alternately with it and with coal a great deal of the heat that now goes up the chimney in the form of smoke will remain to warm the room. If coal alone is to be used, Dr. Bond has a suggestion which would certainly be economical, but which would, we fear, be difficult to adopt with ordinary grates and ordinary chimneys. It is "the construction of the grate in such a way that there are two alternative outlets for the products of combustion into a single chimney: one above the fuel, as in the ordinary grate, for use in starting the fire, so as to heat the chimney, and thus establish a draught; and the other at the bottom of the grate, to be used when the draught has been established *for the purpose of drawing the smoke downwards through the fire, and of thus consuming it.*" He adds that the fire must be fed carefully, so as not to produce a larger amount of smoke than can be readily consumed. For those about to build themselves new houses another suggestion may be given—the putting of the chimneys in the middle of the house instead of on the outside ones. Thus almost all the heat given out by the fires is retained. It is better also to build the fireplaces well forward into the room. The space behind the chimney can be built filled up with shelves for books or bric-à-brac, or turned into a pretty cosy corner. It will be more worthy of the name than many corners so entitled.

For most of us, however, the question is how to make the most of the appliances we have. These may be summarised thus: Always begin the day with a good fire, and after the room has been well warmed replenish the fire frequently, but with only a little fuel at a time; but do not let the fire go down. A large fire well maintained will give more heat and consume less fuel than one allowed to go nearly out and then lavishly renewed. Use coke in combination with coal. Replace iron with firebrick as much as possible in your grates. Most grates are now largely made of brick; but even in an old-fashioned iron grate you may actually add to its heating power, while diminishing its wastefulness, by filling up the back with brick. Bricks at the side lessen the consumption, but also lessen the radiating power of the grate; and always keep control of the draught, so that you can increase or diminish it as the needs of your fire demand.

